# Odorant Receptor (*Or*) Genes: Polymorphism and Divergence in the *D. melanogaster* and *D. pseudoobscura* Lineages

**DOI:** 10.1371/journal.pone.0013389

**Published:** 2010-10-13

**Authors:** Inês C. Conceição, Montserrat Aguadé

**Affiliations:** 1 Departament de Genètica, Facultat de Biologia, Universitat de Barcelona, Barcelona, Spain; 2 Institut de Recerca de la Biodiversitat, Universitat de Barcelona, Barcelona, Spain; American Museum of Natural History, United States of America

## Abstract

**Background:**

In insects, like in most invertebrates, olfaction is the principal sensory modality, which provides animals with essential information for survival and reproduction. Odorant receptors are involved in this response, mediating interactions between an individual and its environment, as well as between individuals of the same or different species. The adaptive importance of odorant receptors renders them good candidates for having their variation shaped by natural selection.

**Methodology/Principal Findings:**

We analyzed nucleotide variation in a subset of eight *Or* genes located on the 3L chromosomal arm of *Drosophila melanogaster* in a derived population of this species and also in a population of *Drosophila pseudoobscura*. Some heterogeneity in the silent polymorphism to divergence ratio was detected in the *D. melanogaster*/*D. simulans* comparison, with a single gene (*Or67b*) contributing ∼37% to the test statistic. However, no other signals of a very recent selective event were detected at this gene. In contrast, at the speciation timescale, the MK test uncovered the footprint of positive selection driving the evolution of two of the encoded proteins in both *D. melanogaster* —OR65c and OR67a —and *D. pseudoobscura* —OR65b1 and OR67c.

**Conclusions:**

The powerful polymorphism/divergence approach provided evidence for adaptive evolution at a rather high proportion of the *Or* genes studied after relatively recent speciation events. It did not provide, however, clear evidence for very recent selective events in either *D. melanogaster* or *D. pseudoobscura*.

## Introduction

Animals can recognise and discriminate chemical signals in the environment, which provides essential information for survival and can profoundly influence their behaviour [1]. In the case of airborne molecules, the recognition starts with their interaction with odorant receptors that reside in the olfactory receptor neurons (ORNs; [2]). These ORNs transmit signals into the Central Nervous System, where they are processed, ultimately leading to behavioural responses.

Odorant receptor (*Or*) genes encode signal-transduction proteins with seven transmembrane domains. In insects, they are members of a large and rather old multigene family, with orthologs in orders as diverse as Diptera, Homoptera, Hymenoptera and Coleoptera (e.g., [3], [4], [5]). Because olfaction contributes to find food and mates as well as to detect predators, genes involved in olfactory perception are candidates to have evolved by the action of positive natural selection. Indeed, a maximum likelihood analysis of nonsynonymous and synonymous divergence across five species of the *melanogaster* subgroup with complete genome sequences revealed that the overall evolution of the *Or* family during the last ∼12 MY was nonneutral [6]. Also, the comparison of *Or* polymorphism in a specieswide sample of *Drosophila simulans* and divergence of those from *D. melanogaster* orthologs provided some evidence of adaptive evolution of OR proteins in the *D. simulans* lineage [6].

The analysis of polymorphism, unlike that of divergence, can uncover the footprint left on DNA sequences by very recent selective events. Moreover, the analysis of polymorphism and divergence at coding regions constitutes a powerful approach to detect the action of recurrent positive selection driving to fixation amino acid changes after relatively recent speciation events. In an effort to uncover the action of positive selection acting on *Or* genes at these two timescales, we have analyzed within-population variation in two well characterized species (*D. melanogaster* and *D. pseudoobscura*) as well as divergence to a closely related species (*D. simulans* and *D. miranda*, respectively) at a subset of eight *Or* genes —*Or63a*, *Or65a-b-c* cluster, *Or67a*, *Or67b*, *Or67c* and *Or69a*— that were solely chosen for their location on the same chromosomal arm of *D. melanogaster* (3L or Muller's D element). In *D. pseudoobscura*, the *Or* genes studied are located on the XR chromosomal arm, with the exception of genes *Or65b2*, *Or65b4* and *Or65b5* that are located on element C (chromosome 3) and gene *Or67a* on element E (chromosome 2) due to transposition events that predated the X-autosome fusion [7]. Our multilocus analysis of polymorphism and divergence provided no clear indication of very recent action of positive selection on the *Or* genes studied. It did, however, uncover the footprint of positive selection driving the evolution of a relatively large proportion of the encoded proteins in both the *D. melanogaster* and *D. pseudoobscura* lineages.

## Results and Discussion

### Levels of polymorphism

Table 1 summarizes the estimated levels of nucleotide variation at the *Or* genes studied in *Drosophila melanogaster* and *D. pseudoobscura*. A total of 18.9 and 19.5 kb were analyzed in each of these species, respectively (Table S1). The number of segregating sites was 445 in *D. melanogaster* and 421 in *D. pseudoobscura*, with the former species exhibiting a lower overall proportion of polymorphic sites with singletons (31%) than the latter species (62%). In both species, the estimated nonsynonymous nucleotide diversity was almost ten-fold lower than synonymous estimates (Table 1). Estimates of noncoding diversity did not differ significantly from those of synonymous diversity in either *D. melanogaster* or *D. pseudoobscura* (Wilcoxon signed-rank test; P = 0.31 and 0.36, respectively), which would seem in contrast with the higher level of constraint at intergenic regions than that at synonymous sites previously observed in *D. melanogaster*/*D. simulans* comparisons [8], [9]. Moreover, similarly to previous surveys [10], [11], [12], no significant difference in the level of either noncoding or synonymous polymorphism was detected in *D. pseudoobscura* between the sex-linked and autosomal genes (Wilcoxon signed-rank test; P = 0.22 and 0.18, respectively). The time elapsed since the X-autosome fusion (8–12 My; [13]) cannot probably account for these results since it would seem sufficient for variation at the newly X-linked arm (XR) to have attained the new equilibrium and therefore for the newly sex-linked genes to exhibit the expected reduction of variation relative to autosomal genes. The previously detected bias in the species sex-ratio toward a higher proportion of females [14] might be one of the factors contributing to the detected similarity.

**Table 1:** Nucleotide variation in different functional regions of the *Or* genes.

			*S*				π				Haplotypes		*K*			
Species	Gene	N^a^	nc	s	a	Total^b^	nc	s	a	Total	No.	*Hd*	*K*_nc_	*K*_s_	*K*_a_	*K*_a_/*K*_s_
*D. melanogaster*	*Or63a*	12	28	14	6	48 (13)	0.009	0.016	0.002	0.007	10	0.97	0.050	0.118	0.011	0.089
	*Or65a*	14	17	17	8	42 (14)	0.005	0.023	0.002	0.006	13	0.99	0.028	0.110	0.023	0.208
	*Or65b*	13	11	4	6	21 (0)	0.023	0.005	0.003	0.006	7	0.83	0.170	0.160	0.033	0.207
	*Or65c*	12	6	34	6	47 (24)	0.014	0.039	0.002	0.011	11	0.99	0.121	0.155	0.034	0.221
	*Or67a*	12	52	17	2	71 (36)	0.011	0.015	0.0004	0.007	12	1	0.106	0.202	0.078	0.388
	*Or67b*	13	20	2	1	23 (4)	0.009	0.004	0.0002	0.004	10	0.95	0.090	0.214	0.009	0.044
	*Or67c*	14	34	26	7	67 (34)	0.006	0.022	0.002	0.006	8	0.82	0.061	0.197	0.009	0.047
	*Or69a*	11	91	23	14	128 (27)	0.023	0.021	0.003	0.014	11	1	0.079	0.119	0.032	0.265
	Total		259	137	50	447 (152)										
	Average						0.013	0.018	0.002	0.008		0.944	0.088	0.159	0.029	0.184
*D. pseudoobscura*	*Or63a*	8	39	9	12	60 (35)	0.039	0.011	0.005	0.014	8	1	0.082	0.065	0.011	0.173
	*Or65b1*	8	21	22	5	48 (26)	0.034	0.028	0.002	0.015	8	1	0.063	0.058	0.024	0.411
	*Or65b2*	7	7	12	7	26 (16)	0.011	0.017	0.002	0.007	7	1	-	-	-	-
	*Or65b4*	6	13	14	17	44 (36)	0.019	0.020	0.007	0.012	6	1	0.048	0.061	0.017	0.285
	*Or65b5*	7	26	21	11	58 (27)	0.013	0.029	0.005	0.012	7	1	0.053	0.115	0.021	0.183
	*Or67a*	8	22	32	12	66 (42)	0.036	0.039	0.004	0.015	8	1	0.078	0.077	0.015	0.199
	*Or67b*	8	7	4	1	12 (6)	0.003	0.004	0.0003	0.002	7	0.96	0.021	0.030	0.005	0.180
	*Or67c*	8	47	24	2	73 (43)	0.011	0.030	0.001	0.009	8	1	0.028	0.040	0.005	0.121
	*Or69a*	8	20	7	7	34 (30)	0.002	0.003	0.001	0.002	8	1	0.033	0.038	0.008	0.224
	Total		202	145	74	421 (261)										
	Average						0.019	0.020	0.003	0.010		0.996	0.051	0.060	0.013	0.222

*S*, no. of segregating sites; *π*, nucleotide diversity; *K*, nucleotide divergence; nc, noncoding; s, synonymous; a, nonsynonymous; *Hd*, haplotype diversity.

a Number of lines sequenced.

b Number of singletons are given in parentheses.

There is evidence of recombination in the history of all genes studied in both *D. melanogaster* and *D. pseudoobscura* (i.e., *R*_m_≥1), with the exception of gene *Or67b* in the latter species (Table S2). As expected from recombination rates based on genetic map distances, the overall degree of genetic association between polymorphisms (as summarized by the *Z*_nS_ statistic; Table S2) was generally higher in *D. melanogaster* (from 0.20 to 0.66) than in *D. pseudoobscura* (from 0.14 to 0.53).

### No clear indication of very recent adaptive substitutions

**Figure 1:**
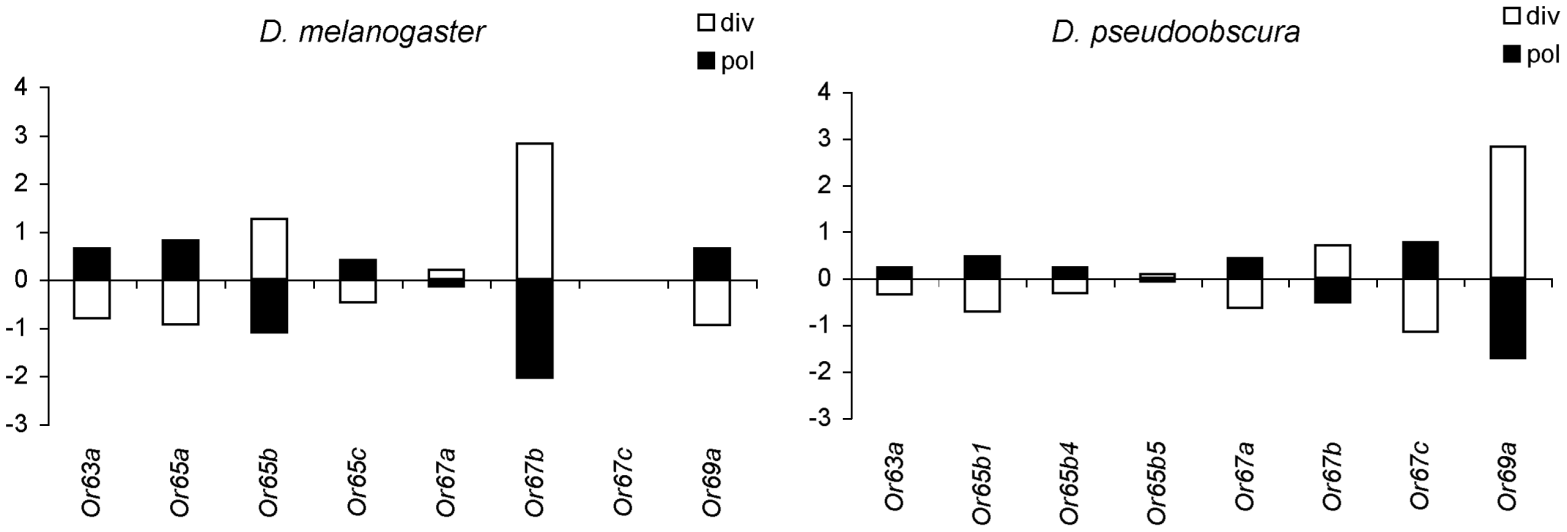
Multilocus HKA. Summary of a multilocus HKA test, which compares polymorphism within *D. melanogaster* and *D. pseudoobscura* to divergence from *D. simulans* and *D. miranda*, respectively. Solid bars represent contributions to the overall χ^2^ test statistic caused by polymorphism levels at each locus; open bars represent contributions caused by divergence. Positive values indicate an excess of polymorphism or divergence relative to neutral expectations. Likewise, negative values indicate a defect relative to expectation.

Multilocus HKA tests were performed using silent polymorphism (in *D. melanogaster* and *D. pseudoobscura*) and divergence (between *D. melanogaster* and *D. simulans*, and between *D. pseudoobscura* and *D. miranda*, respectively; Fig. 1). Only in the *D. melanogaster*/*D. simulans* comparison, the low probability associated to the test statistic (χ^2^ = 13.27; P = 0.07) pointed to a possible decoupling between levels of polymorphism and divergence across genes. In this comparison, a single gene exhibiting a local reduction in polymorphism —*Or67b*— contributed 36.6% to the test statistic. However, no clear signature of a recent selective sweep was detected in the pattern of polymorphism at this gene using either summary statistics based on the frequency spectrum (Tajima's *D* and normalized Fay and Wu's *H* [16], [17], [18]; see below) or the Kim and Stephan test [15], which also considers the spatial distribution of variation (results not shown).

**Figure 2:**
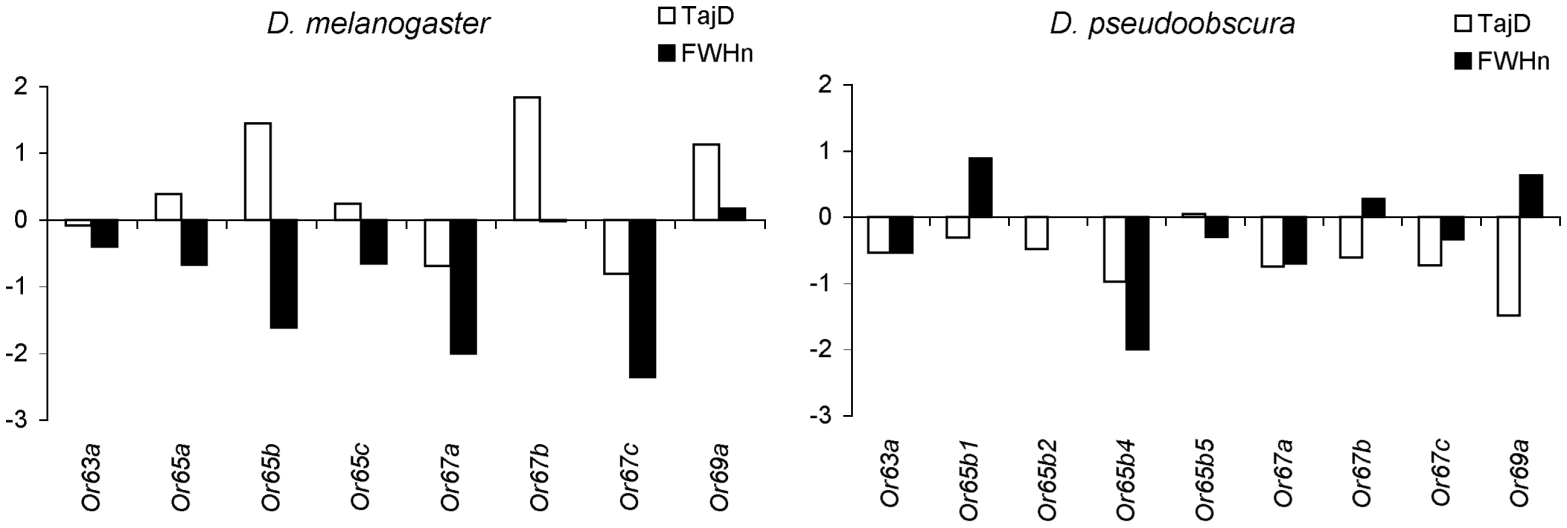
Summary statistics. Tajima's *D* and normalized Fay and Wu's *H* statistics for *D. melanogaster* and *D. pseudoobscura*.

The frequency distribution of nucleotide variants was investigated using Tajima's *D* and normalized Fay and Wu's *H* (Fig. 2; [16], [17], [18]). In *D. melanogaster*, the estimated *D* values varied widely across genes whereas the *H* estimates were generally negative (Fig. 2). The estimated values did not depart from neutral expectations either under stationarity or under the bottleneck scenario proposed for derived European populations ([19], [20], [21]; results not shown).

In *D. pseudoobscura*, a general skew toward negative values of both Tajima's *D* and Fay and Wu's *H* was observed, which resulted in average negative values for both statistics (−0.648 and −0.265, respectively). A similar observation concerning the folded frequency spectrum (i.e., Tajima's *D* statistic) was previously reported in this species and led the authors to consider a scenario of population expansion as the most plausible explanation for the detected pattern [11], [22].

### Evidence for adaptive evolution of ORs in the *D. melanogaster* and *D. pseudoobscura* lineages

The MK test that was performed for each gene separately yielded highly significant results for genes *Or65c* and *Or67a* in the *D. melanogaster*/*D. simulans* comparison, and for genes *Or65b1* and *Or67c* in the *D. pseudoobscura*/*D. miranda* comparison (Table 2). In all these cases, an excess of fixed nonsynonymous changes was detected. When correcting for multiple testing (using the stringent sequential Bonferroni correction; [23]), the tests remained significant for the same four genes. When applying the MK test to the pooled set of genes, highly significant results were obtained in both comparisons (*D. melanogaster*/*D. simulans* and *D. pseudoobscura*/*D. miranda*), indicating a general trend toward an excess of fixed nonsynonymous changes. In all cases, the removal of singleton polymorphisms did not affect the results (results not shown), which together with the below-one values of the neutrality index [24] for all four genes (0.16 and 0.08 for *Or65c* and *Or67a* and 0.15 and 0.06 for *Or65b1* and *Or67c*, respectively) suggests that these genes exhibited indeed a significant excess of nonsynonymous fixed mutations. Moreover, in the *D. melanogaster* comparison, the polarized MK test (using *D. yakuba* as the outgroup) revealed a significant excess of fixed nonsynonymous mutations at genes *Or65c* and *Or67a* in the *D. melanogaster* lineage (results not shown). Little is known about the specific functions of the encoded receptors in each species except that in *D. melanogaster* the receptors encoded by genes of the *Or65* cluster seem to have pheromones as ligands [25] whereas genes *Or67a* and *Or67c* are known to respond strongly to a broad range of food odours [26].

**Table 2:** McDonald and Kreitman tests.

Species	Gene	FS	FNS	PS	PNS	P-value^a^
*D. melanogaster*	*Or63a*	28	8	14	6	0.54
	*Or65a*	24	22	17	8	0.22
	*Or65b*	37	28	4	6	0.50
	*Or65c*	28	30	34	6	<0.001***
	*Or67a*	47	67	17	2	<0.001***
	*Or67b*	52	9	2	1	0.40
	*Or67c*	36	6	26	7	0.54
	*Or69a*	44	47	23	14	0.18
	TOTAL	296	217	137	50	<0.001***
*D. pseudoobscura*	*Or63a*	15	6	9	12	0.12
	*Or65b1*	12	18	22	5	0.003**
	*Or65b4*	7	7	14	17	1.00
	*Or65b5*	24	16	21	11	0.81
	*Or67a*	12	8	32	12	0.39
	*Or67b*	8	5	4	1	0.61
	*Or67c*	3	4	24	2	0.011**
	*Or69a*	18	13	7	7	0.75
	TOTAL	99	77	133	67	0.044*

FS, fixed synonymous substitutions; FNS, fixed nonsynonymous substitutions; PS, polymorphic synonymous substitutions; PNS, polymorphic nonsynonymous substitutions.

*P<0.05;

**P<0.01;

***P<0.001.

a Two-tailed Fisher's exact test.

In both *D. melanogaster* and *D. pseudoobscura*, two of the eight *Or* genes studied exhibited the footprint of protein adaptive evolution. The estimated proportion (0.25 in both lineages) is based on a small number of genes and does not differ significantly from that estimated (0.1) in a genomewide study, which included a larger number of *Or* genes (20) that were partially sequenced in a sample including both African and cosmopolitan lines of *D. melanogaster* [27]. This relatively high proportion would seem consistent with diverse observations in *D. melanogaster*. Indeed, in this species the expression of some chemoreceptor genes is highly sexually dimorphic and frequently sexually antagonistic, and the extent of transcriptional responses to changing conditions is heterogeneous among the chemoreceptor repertoire [28]. Moreover, some of the encoded proteins have indeed pheromones as ligands and they might either signal the presence of inappropriate mating partners or contribute to the identification of conspecific partners [29]. Other odorant receptors exhibit a strong response to food odours and might serve to signal food sources in the environment. The challenges imposed by changing environmental conditions, such as those often associated with speciation and species range expansions, might thus trigger the adaptive evolution of ORs and also promote adaptive regulatory changes in the chemoreceptor genes. However, the proportion of *Or* genes under positive selection detected in both our study and the genomewide study [27], as well as that of *Gr* genes (2 out of 20) in the latter study, do not differ significantly from the proportion of non-chemosensory genes (29 out of 379; [27]). A similar result was obtained when chemosensory (*Or* and *Gr*) genes in *D. simulans* [6] were compared to a genomewide sample of non-chemosensory genes [30]. In Drosophila, adaptive protein evolution at the speciation timescale —as evidenced by the polymorphism to divergence comparison in *D. melanogaster*, *D. simulans* and *D. pseudoobscura*— would thus seem as pervasive among ORs as among the rest of proteins.

## Materials and Methods

### Drosophila strains

Fourteen isochromosomal lines for the third chromosome of *Drosophila melanogaster* obtained from a natural population of Sant Sadurní d'Anoia (Spain; [31]), and 13 highly inbred lines of *D. pseudoobscura* from a natural population of Davis (USA; kindly provided by C. Segarra) were used for the analysis of polymorphism. Highly inbred lines obtained by ten generations of sib-mating were also used for the analysis of divergence: one line each of *D. simulans* (Mozambique; [32]) and *D. miranda* (kindly provided by C. Segarra).

### DNA extraction, amplification and sequencing

DNA was extracted from i) one single individual per inbred line (a male in the case of *D. pseudoobscura* and *D. miranda*); and ii) ten individuals per isochromosomal line, using either a modification of protocol 48 in Ashburner [33] or the PUREGENE DNA Purification kit (Gentra Systems, Inc.) for DNA extraction of a single fly.

Amplification and sequencing primers were designed based on the *D. melanogaster* and *D. pseudoobscura* genome sequences using program Oligo 4 (Molecular Biology Insights, Inc.). In general, amplification primers were designed to be conserved between species. Sequencing primers were species-specific and spaced on average 500 nucleotides. The purification step was a modification of the protocol described in Dean *et al.* [34]. Sequencing products were ethanol precipitated and later separated on automatic sequencers ABI 377 or ABI 3700 (ABI Applied Biosystems). All sequences were obtained on both strands. The sequences reported in this article are deposited in the EMBL sequence database library under accession numbers EU274289, EU128651 and FR669264 – FR669446.

### Sequence Analysis

For newly generated sequences, consensus sequences were obtained using the SeqMan program of the DNASTAR Lasergene software package [35]. *Or* genes from *D. yakuba* were downloaded from the Comparative Assembly Freeze 1 (CAF1), according to the GLEANR Annotation in the AAAWiki website (http://rana.lbl.gov/drosophila/; [36]). Sequences were aligned using the MegAlign program of the DNASTAR Lasergene software package [35] or the BioEdit program [37].

The MacClade program [38] was used to edit the DNA alignments for further analysis. Most analyses of polymorphism and divergence were performed using the DnaSP program [39]. The normalized Fay and Wu's *H* statistic [18] was calculated with a program kindly provided by S. E. Ramos-Onsins.

The level of DNA polymorphism was estimated as the per-site nucleotide diversity (*π*: [40]), and nucleotide divergence between species as *K*, the number of per-site substitutions corrected according to Jukes and Cantor [41]. The minimum number of recombination events (*R*_m_) was calculated according to Hudson and Kaplan [42]. The *Z*_nS_ statistic [43] was used to quantify the overall genetic association (linkage disequilibrium) between polymorphic sites.

Four tests were used in order to detect the footprint left by recent selective events on the level and pattern of polymorphism: the Hudson-Kreitman-Aguadé test (HKA test: [44]), the Tajima's *D* [16] and the normalized Fay and Wu's *H* [17], [18] tests, and the maximum likelihood Kim and Stephan test [15]. The multilocus HKA test was conducted using program HKA (distributed by Jody Hey through http://lifesci.rutgers.edu/~heylab). Moreover, the McDonald and Kreitman test (MK test; [45]), which compares the ratios of nonsynonymous to synonymous polymorphic and fixed changes was used to detect the footprint left by recurrent positive selection acting at the protein level after speciation.

## Supporting Information

Table S1 Number of nucleotide positions.
(0.01 MB PDF)

Table S2 Genetic association.
(0.01 MB PDF)
