# Supplementary material for: Odorant Receptor (Or) Genes: Polymorphism and Divergence in the D. melanogaster and D. pseudoobscura Lineages
Source: PLoS One. 2010 Oct 13;5(10):e13389. doi: 10.1371/journal.pone.0013389 (PMC2954185; doi:10.1371/journal.pone.0013389)
Supplement: Table S1 — Number of nucleotide positions. (0.01 MB PDF) [file pone.0013389.s001.pdf]

Table S1. Number of nucleotide positions

Species	Gene	No. of sites[a]			
		**nc (intron)**	**s**	**a**	**Total**
D. melanogaster	*Or63a*	937 (616)	297	963	2197
	Or65a	1054 (236)	282	969	2305
	Or65b	219 (219)	276	942	1437
	Or65c	232 (232)	283	947	1462
	Or67a	1465 (190)	277	944	2686
	Or67b	1094 (517)	285	978	2357
	Or67c	1494 (174)	271	941	2706
	Or69a	1676 (210)	481	1622	3779
	Total	8171 (2394)	2452	8306	18928
D. pseudoobscura	*Or63a*	364 (364)	274	803	1444
	Or65b1	231 (231)	278	868	1379
	Or65b2	224 (224)	298	962	1484
	Or65b4	239 (160)	268	857	1364
	Or65b5	793 (228)	296	952	2041
	Or67a	182 (182)	297	966	1447
	Or67b	912 (486)	296	964	2172
	Or67c	1366 (188)	293	925	2584
	Or69a	3431 (206)	515	1642	5588
	Total	7742 (2274)	2815	8939	19503

nc, noncoding sites; s, synonymous sites; a, nonsynonymous sites. [a] Excluding sites with alignment gaps.
