# Supplementary material for: Odorant Receptor (Or) Genes: Polymorphism and Divergence in the D. melanogaster and D. pseudoobscura Lineages
Source: PLoS One. 2010 Oct 13;5(10):e13389. doi: 10.1371/journal.pone.0013389 (PMC2954185; doi:10.1371/journal.pone.0013389)
Supplement: Table S2 — Genetic association. (0.01 MB PDF) [file pone.0013389.s002.pdf]

Table S2. Genetic association

Species	Gene	R_M	C_h	C_{map}	Z_{nS}
D. melanogaster	*Or63a*	10	0.005	0.06	0.20
	Or65a	7	0.01	0.08	0.29
	Or65b	1	0.000	0.08	0.66
	Or65c	7	0.02	0.08	0.31
	Or67a	8	0.003	0.07	0.21
	Or67b	1	0.003	0.07	0.52
	Or67c	4	0.001	0.06	0.33
	Or69a	13	0.01	0.05	0.27
D. pseudoobscura	*Or63a*	14	2.10	0.37	0.14
	Or65b1	11	0.09	0.37	0.20
	Or65b2	2	0.02	0.22	0.31
	Or65b4	3	0.00	0.22	0.42
	Or65b5	6	0.02	0.22	0.39
	Or67a	11	0.06	0.33	0.22
	Or67b	0	0.21	0.37	0.53
	Or67c	11	1.18	0.30	0.15
	Or69a	2	0.09	0.30	0.15

R_M, minimum number of recombination events in the history of the sample; C_h, recombination parameter per nucleotide according to (Hudson 1987); C_{map}, recombination parameter based on map distances.
